# Supplementary material for: Humans as geomorphic agents: Lidar detection of the past, present and future of the Teotihuacan Valley, Mexico
Source: PLoS One. 2021 Sep 20;16(9):e0257550. doi: 10.1371/journal.pone.0257550 (PMC8452071; doi:10.1371/journal.pone.0257550)
Supplement: S1 File — (PDF) [file pone.0257550.s001.pdf]

# S1 File. Additional Tables

| Feature Type | N |
|---|---|
| Mounds | 7 |
| Depressions | 6 |
| Terraces | 192 |
| **Total** | **205** |

**S1A Table. Quantity of features affected by mining for each feature type.**

| Type | Inside Grid | Outside Grid | Grand Total |
|---|---|---|---|
| Depression | 33 | 136 | 169 |
| Mound | 592 | 138 | 730 |
| Plaza | 57 | 10 | 68 |
| Structure | 250 | 14 | 264 |
| Terrace | 519 | 5,795 | 6,314 |
| **Total** | **1,451** | **6,093** | **7,544** |

**S1B Table. Quantity of features by type inside and outside the Millon Grid.**

| Feature Type | Total Count | Ground Verified | Percent of Total |
|---|---|---|---|
| Depression | 99 | 26 | 26% |
| Mound | 67 | 40 | 60% |
| Plaza | 7 | 5 | 71% |
| Structure | 5 | 4 | 80% |
| Terrace | 4,409 | 2,020 | 46% |
| **Total** | **4,587** | **2,095** | **46%** |

**S1C Table. Count and ground verification status of confidence level 1 or 2 features outside of Millon Grid.**

| Heat Map Categories - Combined | Grid Square Count | Percent of Total |
|---|---|---|
| 0 $m^2$ | 14,104 | 71.1% |
| 1 - 50 $m^2$ | 1,659 | 8.4% |
| 51 - 100 $m^2$ | 1,023 | 5.2% |
| 101 - 200 $m^2$ | 1,328 | 6.7% |
| 201 - 2200 $m^2$ | 1,711 | 8.6% |
| **Total** | **19,825** | **100.0%** |

**S1D Table. Count and percentage of 100m grid squares for each length category of Teotihuacan alignments (.**

| Description | Count |
|---|---|
| Grid squares w/ no alignments | 14,104 |
| Grid squares w/ alignments | 5,721* |
| **Total 100m grid squares** | **19,825** |
| Grid sq w/ centroid in urban areas | 3,910 |
| Grid sq w/ alignments & centroid in urban areas | 2,545** |
| **Total 100m grids in urban areas** | **6,455** |

**S1E Table. Counts of grids with Teotihuacan alignments in general, and within urban zones in particular.** *29% of 100m grids contain Teotihuacan alignments, **65% of urban grids contain Teotihuacan alignments.

| Label | $Km^2$ | Percent |
|---|---|---|
| Ancient - Hydrologic | 0.2 | 0.1% |
| Ancient - Subsurface | 33.0 | 20.1% |
| Ancient - Excavated | 2.5 | 1.5% |
| *Ancient Total* | *35.6* | *21.6%* |
| Mixed - Hydrologic | 0.7 | 0.5% |
| Mixed - Subsurface | 4.7 | 2.8% |
| *Mixed Total* | *5.4* | *3.3%* |
| Modern - Hydrologic | 0.2 | 0.1% |
| Modern - Urban/Built | 21.0 | 12.8% |
| Modern - Agricultural | 78.0 | 47.4% |
| *Modern Total* | *99.2* | *60.3%* |
| Natural - Hydrologic | 0.4 | 0.3% |
| Natural - Forest | 2.3 | 1.4% |
| Natural - Grassland | 9.0 | 5.5% |
| Natural - Scrubland | 12.5 | 7.6% |
| *Natural Total* | *24.3* | *14.8%* |
| Total | 164.4 | 100.0% |

**S1F Table. Area and percent area of each land use type.**
