# Supplementary material for: Humans as geomorphic agents: Lidar detection of the past, present and future of the Teotihuacan Valley, Mexico
Source: PLoS One. 2021 Sep 20;16(9):e0257550. doi: 10.1371/journal.pone.0257550 (PMC8452071; doi:10.1371/journal.pone.0257550)
Supplement: S2 File — (PDF) [file pone.0257550.s002.pdf]

S2 File. Methodologies

Lidar data acquisition and processing

The National Center for Airborne Laser Mapping (NCALM) partially based at the University of Houston, Texas collected this lidar dataset using at the time the new Teledyne Optech Titan MW system [1], which contains significant improvements from the sensors used in earlier projects [2–5]. This multispectral lidar sensor collects data at 532, 1064, and 1500 nm wavelengths (each capable of pulse repetition frequencies of 50-300 kHz), and can record full waveform data to supplement the standard discrete returns stored into the standard LAS file format [6]. Data collection for 165 km² took place on April 1st and 2nd, 2015, flight lines were planned with a lateral spacing of 450 m and 900 m above the ground level with an aircraft speed of 77 m/s (~150 knots). The sensor was configured with a PRF of 250 kHz per channel (750 kHz total) and a scanner oscillating at 20 Hz in a ± 30º range. Lidar data were collected along swaths a km wide while overlapping half of the swath with the adjacent ones (50% lateral overlap). The combination of above parameters produced a nominal lidar pulse density of 15 pulses/m², however additional flight lines along the Avenue of the Dead and other areas produced an average density of 18.4 pulses/m².

The total number of processed pulsed for the project was 3.089 billion, which produced 3.218 returns, or 1.04 returns per pulse reflective of the overall low vegetation coverage in the area. The vertical precision of the lidar returns was assessed against 581 geodetic grade kinematic GPS checkpoints yielding 4.1 cm and the horizontal precision is estimated a 15-20 cm (both at one sigma).

Lidar processing was performed through NCALM procedures [7]. Point cloud was classified into three basic classes using automated algorithms running in Terrasolid TerraScan software with some minimum manual editing, these classes are a) ground including ancient buildings and structures; b) non-ground which includes vegetation, modern building and infrastructure, vehicles, etc; and c) outliers such as returns from flying birds and extremely long returns. The classified LAS files were interpolated into digital elevation models (DEM) employing Kriging implemented as scripts in Golden Software Surfer. Given the high ground return density of this dataset, with the 25 to 75 percentiles in the range of 7.4 to 11.9 ground returns per m² it was possible to generate the DEM at half-meter raster spacing (four times the resolution of most other projects). For lidar product interpretation purposes, both the bare-earth (i.e. defoliated) and a digital-surface-model (i.e. fully vegetated) DEMs have been used for analysis to reduce the digitizing of false-positive features that may have been introduced through classification and Kriging interpolation, while using various visualization algorithms.

Enhanced DEM visualizations

Human identification of features in this project's DEMs has benefited from the use of various visualization methods to fit the needs of the features to be identified and the landscape. We employed and tested hillshaded terrain models simulating raking light on the landscape [8], principle components analysis of those hillshades from multiple lighting angles [9], slope models

using various color ramps [10], local relief models to highlight small features on the landscape [11,12], and sky-view factor visualizations to highlight walls [13,14]. However, the sky-view factor and slope model visualizations has become the primary visualization method used by project members due to the relatively gentle slopes present on the landscape around Teotihuacan. Newer composite visualization methods may also hold promise for future investigations [15].

Human Visual Feature Identification

The lidar data was hosted on ArcGIS Online, a cloud-based mapping platform that is used for the collection, management, and sharing of spatial data [16]. This technology allowed students in the United States to identify features on the lidar-derived models, while the ground crew in Teotihuacan, Mexico worked on ground verification. Confidence level designation pre-ground verification and post-ground verification allowed the team to periodically assess the accuracy of lidar-based feature identification, and also ensure all team members had access to the most current version of the data, reducing errors and saving time. The online map was also linked to the ESRI Collector [17] smartphone app which the field crew used to capture, edit, and reference data layers during ground reconnaissance assessments. The app's base maps and imagery helped communicate project goals to private landowners, and seek their permission to investigate features on their property. Each week team members from up to three time zones hold a virtual meeting via WhatsApp [18] to discuss discrepancies between each of their work and refine feature detection standards. Several times throughout the year, the features are tallied, and a review of the total feature counts and feature identification accuracy is conducted. This review determines how to adjust the focus of feature detection and field work. Below we describe each feature type identified as separate shape files in the Teotihuacan Valley Lidar database.

Though many features were detected through this process (Table 1), we found the lidar map significantly under-represented those proposed through traditional survey. There were 3,434 individual structures in Millon's map that we digitized while we record only 1,061 mounds/plazas/structures, accounting for only 31% of the total count within Millon's surveyed area. When examining overlap of lidar features in comparison to Millon's hypothetical structures with a 20 m buffer (accounting for difference in accuracy and scale between Millon's map and our lidar map), only 328 features roughly overlapped with Millon's structures (10%).

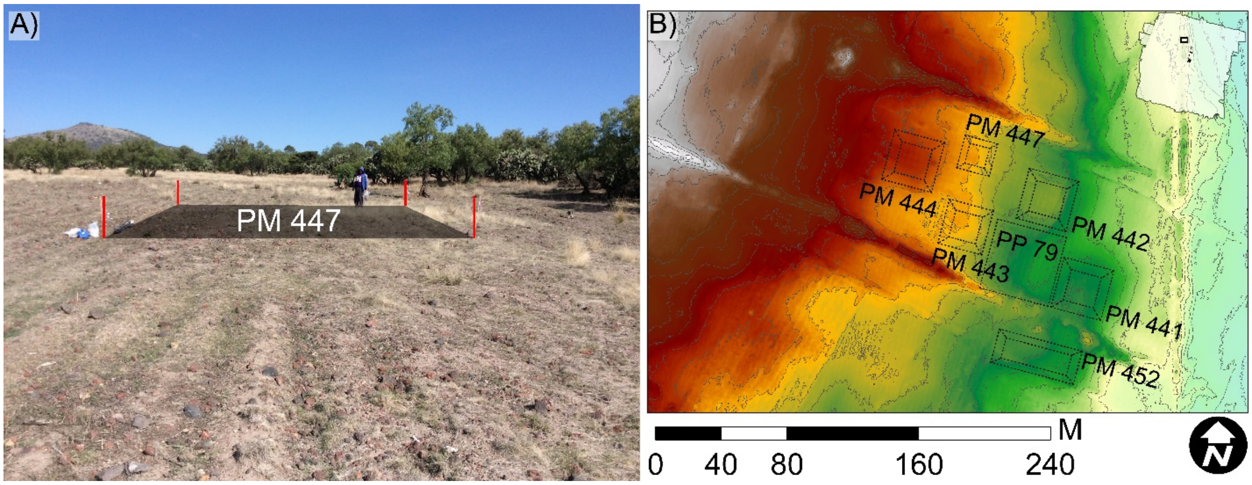

S2A Fig. Examples of mounds and a plaza in ejido de Purificación-Oztoyahualco.
A) photograph from ground verification survey of PM 447, and B) lidar DEM with 0.5 m contour lines of area indicating six potential mounds and a plaza (PP 79).

Mounds: Potential mounds are identified via a topographic rise. They may include platforms, structures, and other subsurface features but often cannot be defined to further detail from the surface. During ground reconnaissance each feature's temporal assessment was determined from surface collections. Many historically surveyed mounds were no longer detected on the lidar map and confirmed only through artifact scatters found during ground survey. Often, 0.5m contour lines were necessary to assess subtle topographic shifts as extensive agricultural practices have substantially flattened these mounds (Fig S2a).

Structures: Structures are characterized by visible wall alignments (Fig 3C). We often referenced Millon's potential structures to verify any topographic characterizations that led to their placements. These features tended to concentrate only within Millon's map area. Ground reconnaissance usually required verification of the alignment and a temporal designation based on surface collection, as many historic structures tended to leave more exposed visible wall alignments distinct from mounds.

Plazas: Plazas are underrepresented because they are defined by their flatness and there are many causes for land levelling. Plazas were primarily recognized by their association to surrounding features, usually by their typical three-temple complex layout (e.g. Fig S2a, PP 79). Plazas tend to be cleaned after usage, and later extensively reutilized for cultivation, resulting in low artifact scatter.

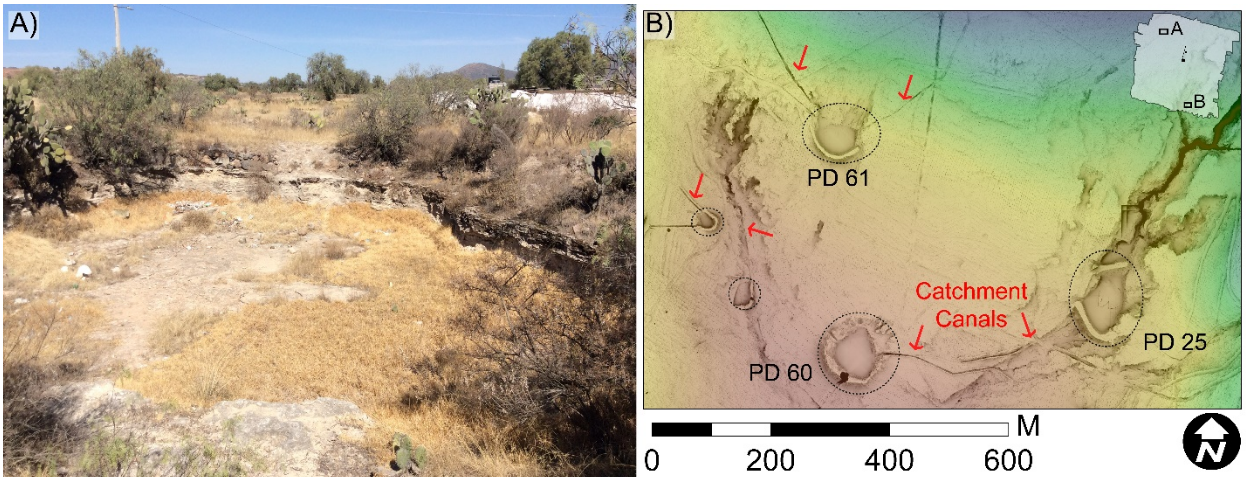

S2B Fig. Two depression examples.
A) photograph of PD 66 in Tlachinolpa, and B) four depressions found on the lidar DEM on the south sector of Sierra Patlachique. Note: other two depressions in B) are modern and were not given a feature number.

Depressions: Natural and cultural depressions include river courses, water reservoirs, and mines, often found in close proximity to cultivated fields. Excavation typically intrudes into the bedrock that is connected to a water run-off. It is difficult to date these features, as there has been little to no change in the techniques used to create them prior to the industrial era. Ancient handcrafted water features tend to be diverse shapes and sizes (Fig S2b) while those employing modern machinery are symmetrical and round or ovular.

Terraces: As reported by Sanders [19], the hillsides in the Teotihuacan Valley are heavily terraced. Once established terrace systems tend to be utilized perpetually and continuously, complicating chronological assessment. Terraces were the most common feature type we surveyed yet, our accuracy remains very low, between 15-20% (Table 2). We observed a distinction between narrower old terraces that follow the contours of the topography and modern terraces that are wider and rigid (Fig 3E). Local knowledge by village elders recounted some of the stone wall terraces were built by agricultural laborers for Haceinda Actipac during the XIX-XX centuries (Fig S2c). Ground reconnaissance allowed us to reassess some features originally identified as terrace lines as modern alterations resulting from reforestation efforts. These reforestation works follow a sharp double line pattern which conforms to the natural topographic curve of the hillside.

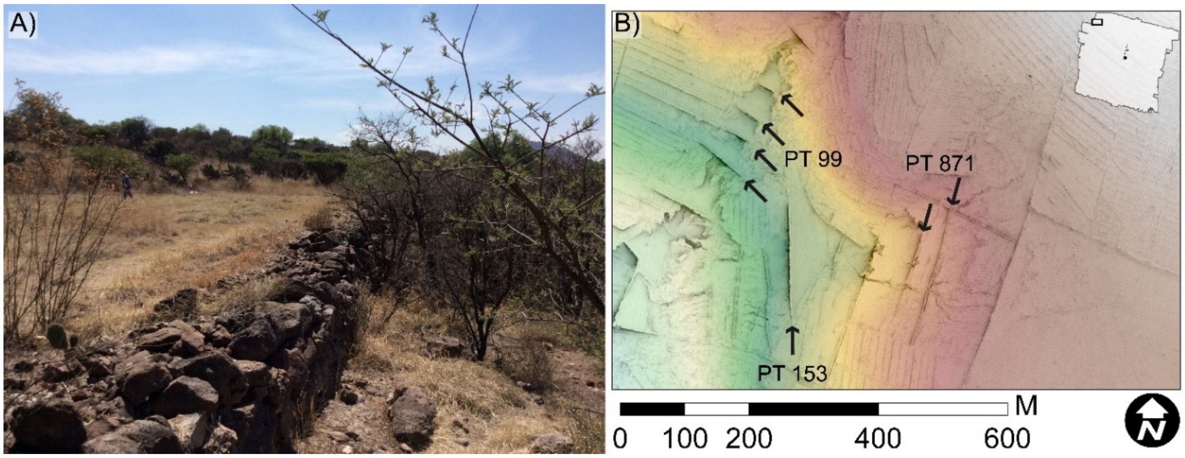

S2C Fig. Example of hacienda period terrace walls from San Augstín Actipac.
A) Photograph taken during ground reconnaissance and b) on a colored shaded relief map generated from the lidar DEM.

Artifact scatter: In some instances, extensive artifact scatter was found despite the lack of topographic features. We found many areas where Millon registered potential structures had no topographic indicators, and often artifact scatters were the only indication of possible subsurface features. Artifact scatters tended to be located in high erosion zones or along the edge of a hill. With many hilltop sites completely eroded (e.g. Altica), it is important to report areas of high artifact scatter that may comprise the only remaining archaeological vestiges. In total 32 artifact scatter entries covering 0.15 km^2 were recorded from ground reconnaissance. These can be considered lidar omission errors, though a more systematic survey of areas with no features detected will be necessary to generate a more accurate omission rate. We suspect given the abundant instances of no features detected along Millon's hypothetical structures, this omission rate is significantly higher than most quantities reported in the Maya region (e.g. Inomata et al. 11-33% omission error) [20].

Millon discrepancy: This shape file reports 22 areas where excavations or other topographic features have verified a discrepancy between Millon's original map. These features should refine Millon's map with more accuracy as we continue to georeference more detailed maps from excavations that reveal subsurface features.

Modern property alignment: Many modern streets and land boundaries still follow Teotihuacan's 15°east of astronomical north. In order to better understand this phenomenon, the team used modern property boundary data known as "manzanas" created in 2015 by Mexico's National Commission for the Knowledge and use of Biodiversity (CONABIO) [21] and also cadastral data created in 2018 by Mexico's Office for Agrarian, Land and Urban Development (SEDATU) [22] to locate modern aligned property boundaries. Layers called, "Manzanas por localidad, Marco Geoestadistico 2015" in the former maps contained boundaries that represent larger groups of residential and commercial lots that supplemented the cadastral data in areas where cadastral data did not exist. The cadastral data contains more precise boundaries for individual lots of land but was not available for the entirety of the area covered by the lidar data. This data was provided through a web map service, which allows users to view the data but not interact or

manipulate the individual records. In order to use it in GIS, sections of the data were exported as a series of .png files in ArcMap. The ArcScan [23] extension was used to convert the .png files into a single polyline layer.

Modern Teotihuacan alignment: To account for any buildings, structures, or dirt roads that may follow the Teotihuacan orientations which were not captured by the manzanas or cadastral datasets, we relied on manual feature detection and digitization using both the DSM from the lidar map and satellite imagery. Modern Teotihuacan alignments filled important gaps in defining modern alignments in some non-urbanized zones, including most notably the area within the Teotihuacan Archaeological Park where property alignments do not occur. Each modern Teotihuacan alignment was categorized by the feature type; paved road (green); non-concrete road or path (red); natural or man-made boundary (often rock piles) (blue); structures, including all permanent architectural features (buildings, green houses, patios, etc.) (yellow); and other (e.g. archaeological features, parking lots, sports fields, and cemeteries) (grey) (Fig 4). For this step, we digitized any modern features that were not contained in the first two layers if they were reasonably believed to be within a margin of the Teotihuacan alignment (±5°).

Creating heat maps of Modern Property Alignment and Modern Teotihuacan Alignment: The modern property alignment and the modern Teotihuacan alignment datasets were eventually combined into a single layer representing all features potentially aligned to 15°N. The final layer was split into small segments of 100m in length or less, and a new attribute called "Angle" was added. A Python script calculated the angle of each line segment in degrees. All lines within 5° of the 15°N alignment, or perpendicular, were identified as "Aligned". We initially chose to use a broad +/-5 degree as our limit, since we were unsure how well individual features would align to 15°N, or how much error would result from our own manual digitizing of these features. We then sorted the data based on whether line segments were within 5, 4, 3, 2 or 1 degree of 15°N, to see if there was a significant drop in numbers at any point which there was not (Fig S2d, Table S2a).

	5deg	4deg	3deg	2deg	1deg
**Modern Property Alignments**					
meters	837,995.72	705,516.40	553,811.70	402,486.68	243,782.64
% change	NA	(0.16)	(0.22)	(0.27)	(0.39)
cumulative change	NA	(0.16)	(0.34)	(0.52)	(0.71)
**Modern Teo Alignments**					
meters	929,885.79	783,091.37	617,034.03	424,109.23	213,441.42
% change	NA	(0.16)	(0.21)	(0.31)	(0.50)
cumulative change	NA	(0.16)	(0.34)	(0.54)	(0.77)
**Combined Alignments**					
# of grids w/ alignments	7,290	6,520	5,721	4,827	3,663
% total grids w/alignments	37%	33%	29%	24%	18%

S2A Table. Combined Teotihuacan alignment feature lengths for each degree of error.

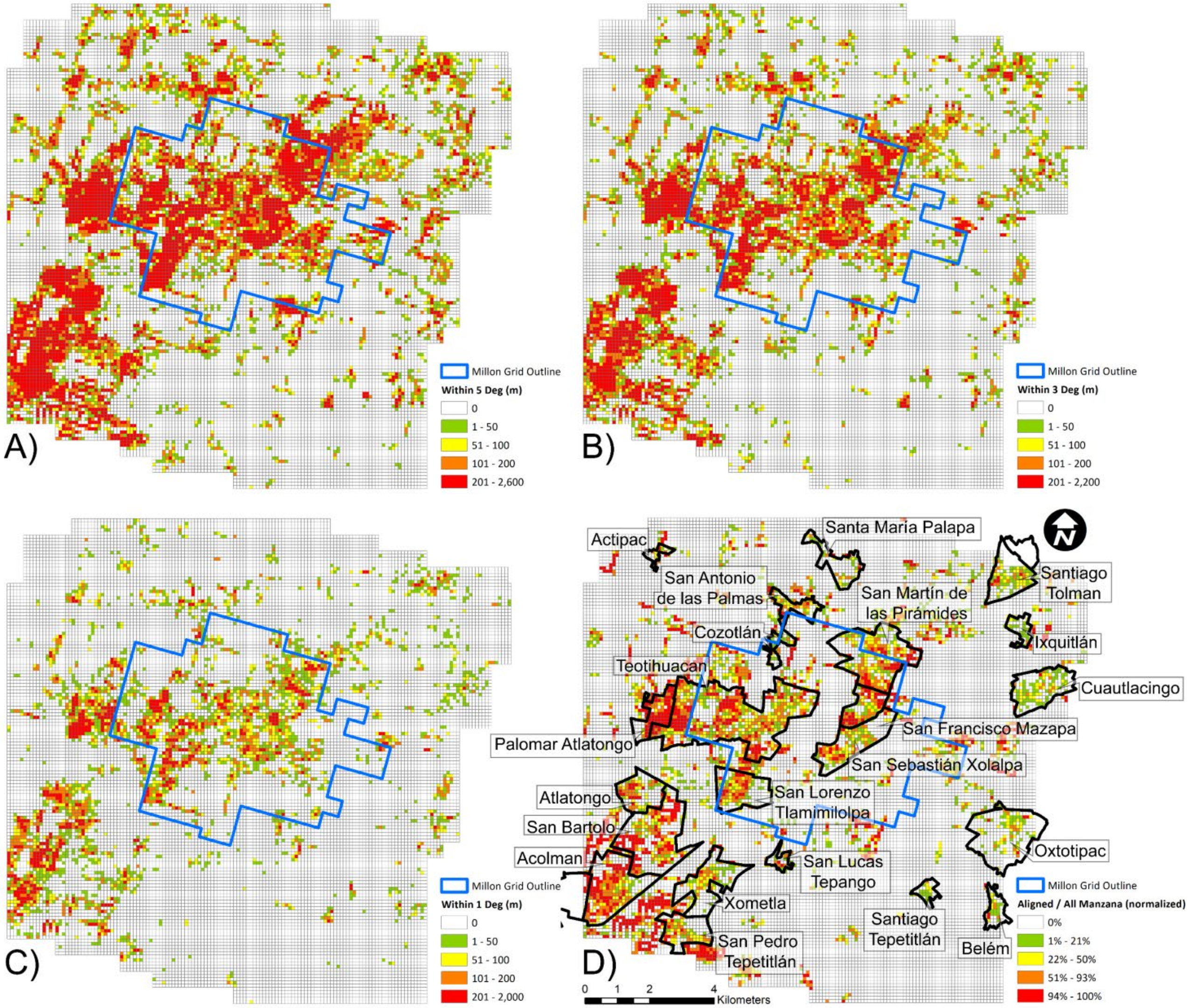

S2D Fig. Heat maps of combined Teo Modern Alignment and Manzana data.
A) 5 degrees error range, B) 3 degrees error range, C) 1 degree error range, and D) the distribution of Modern Property Alignment layer normalized (5 degrees) that highly correlated with urban limits.

We then created a new grid containing 100m x 100m squares, based on the original 500m survey grid. For each grid square, we calculated the total length of aligned line segments and created a heat map for all features within 5, 3, and 1 degree. The heat maps allowed us to identify areas with high concentrations of aligned features for further investigation. Again, we found that the patterns of concentration remained the same between 5, 3, and 1 degree. Based on this analysis, we chose to reduce our limit and only work with lines that were at least +/- 3 degrees of 15°N, to be sure to focus on features that were more likely impacted by the ancient landscape. The final layer was incorporated into the ArcGIS Online map and was used as a guide while analyzing the lidar models. This allowed the team to identify areas where there was a concentration of modern aligned features where there was a high likelihood of finding ancient features.

Lidar (GIS) and Total Station (CAD) Data Integration

Lidar data was produced in reference to the WGS-84 ellipsoid and projected to UTM zone 14N. The total station CAD data was referenced to the 1962 Millon grid. To derive the horizontal (XY / EN) transformation between the UTM 14N and the Millon coordinates 29 reference points were chosen for which the coordinates in both systems is known and well defined (clear corners). From these coordinates the parameters (Dx, Dy, M, R) for two 2D Helmert transformation were determined using Terrasolid Terrascan. These transformations allow for conversion between the two reference systems. The root mean square error (RMSE) of the residuals of the transformation are roughly 55 cm in each orthogonal direction and a combined 2D RMSE of 77.5 cm, which are just about the size of a single grid cell (pixel resolution) in the digital elevation model.

For accurate elevation differences and volume computation utilizing both the lidar and total station data it was decided to adjust the lidar data to match the total station vertical reference. The first step in this process was to convert the lidar ellipsoidal elevations into orthometric elevations employing a geoid model. Two models that were considered are the global Earth Gravitational Model 2008 (EGM08) and the Geoide Gravimetrico Mexicano 2010 (GGM10). Both models have similar horizontal spacing but it was found that the GGM10 capture better the complexity of the geoid ondulations in the study area. After the geoid adjustment, 52 elevation check points obtained from flat uniform surfaces were used to determine and compensate for offset between the total station and the orthometric lidar elevations. From these check points it was determined a relative mean offset of 1.193 m with a standard deviation of 0.112 m. The offset was removed from the lidar elevations to make these directly comparable to the total station ones. The standard deviation is an estimate of the uncertainty of the combined lidar and total station elevations.

Artificial Ground Volume Calculation

In order to ensure the accuracy and precision of our volume calculations, we used a multimethod research approach by employing two different GIS software programs, ArcGIS Desktop 10.7 and Golden Software Surfer 16. Both of these programs compute the volume between the final adjusted lidar surface models as a top surface, and interpolated bedrock models

as a bottom surface and structure outlines to define the horizontal limits for the computation. Three different bedrock models were computed for each volume computation area based on a total of 4464 bedrock elevations obtained with total station, many of which were mapped based on primary excavation data. The models were interpolated using Empirical Bayesian Kriging (EBK) in ArcGIS, triangulation with linear interpolation (TLI) and basic Kriging in Surfer. TLI is the most basic method to generate a surface model from a set of irregularly spaced elevation points, Kriging is the calculation of spatial dependency between pairs of points in a dataset, which results in a statistical model called a semivariogram. EBK is an advanced form of kriging that attempts to reduce the amount of error in this model by repeating the process multiple times. For each new model, a probability is calculated for each point to show the likelihood that the observed data could have been derived from the model [24]. The results can then be quantitatively examined and compared to ensure its accuracy.

Once the bedrock surfaces were created, we then used both software programs to calculate the difference in volume between bedrock and the adjusted lidar models bounded horizontally by a perimeter files for each front in the project area. In ArcGIS, this was accomplished using the "Cut/Fill" tool which compares two coincident elevation layers to produce a new raster indicating height differences between them. In Surfer 16 the Grid Volume tool was employed which provide an extensive report for the volumes and areas including cut, fill and net volume computed with the trapezoidal formula and the net volume computed with the Simpson and Simpson 3/8 formulas. The main difference between the ArcGIS and Surfer is that for the Surfer tool to work the top and bottom surface models have to be of the same extent and spacing, while with ArcGIS computation can be done with models of different extents and spacing. Table S2b presents volume computation results comparisons for four structures using ArcGIS and averaged values from the three named formulas above. For Surfer 16, these differences are less than 2% and are due to slight differences in the implementations of the volume computation algorithms and the surface resampling necessary to be able to apply the volume computation formulas (Table S2b). The values in Table S2b are just for software comparison reference and do not represent our final volume estimates.

The volume values reported in the main manuscript (Text and Table 3) are averaged values of all the computed volumes within Surfer 16 using the three different methods (trapezoidal, Simpson and Simpson 3/8) and the three different bedrock methods (TLI, Kriging and Empirical Bayesian Kriging) (Table S2c). We consider this averaged value represents the most accurate estimate minimizing the uncertainty of each imperfect data source (lidar, total station). The uncertainty values are estimated by expanding or contracting the length and widths of the structures based on the root mean square error (RMSE) of the residuals (55 cm) from the control points that were used for the horizontal transformation of the lidar and total station references; and vertically by the combined uncertainty of the lidar to total station adjustment (11 cm) and the vertical uncertainty of bedrock modeling computed as the standard deviation of the volume estimates divided the structure footprint area.

Structure	ArcGIS Vol m^3	Average Surfer Vol m^3	Difference m^3	Difference %
Sun Pyramid	1,253,069	1,250,754	2,315	0.2%
Moon Pyramid	416,423	414,326	2,097	0.5%
Citadel*	844,753	854,997	10,244	1.2%
PPCC	370,828	377,019	6,191	1.64%

S2B Table. Comparison of the volume calculations between ArcGIS and Surfer methodologies employing the EBK modeled bedrock elevations.
* During the analysis of different volume computations, it was determined that EBK modeling of the bedrock under the Citadel introduced significant error in the volume estimate reported above. Thus, all the volume reported in the main text and in this supplement for the Citadel ignore the values derived from using the EBK bedrock model.

	Volume m^3				+/- Uncertainty	
Structure	Trapezoidal Rule	Simpson's Rule	Simpson's 3/8	Average	m^3	%
Sun Pyramid	1,249,933	1,249,954	1,249,924	**1,249,937**	5,876	0.5
Moon Pyramid	412,084	412,078	412,072	***412,078***	3,136	0.8
Citadel	761,400	761,429	761,358	***761,396***	18,217	2.4
PCC	372,033	372,107	372,028	***372,056***	16,944	4.6

S2C Table. Volume and degree of uncertainty for the Sun Pyramid, Moon Pyramid, Citadel, and Plaza of the Columns Complex (PCC).
Averaged Surfer volume calculation based on Trapezoidal, Simpson's, and Simpson's 3/8 Rules using three different bedrock models (except for the Citadel for which the EBK model was not included in the computation).

References Cited

1. Fernandez-Diaz JC, Carter WE, Glennie C, Shrestha RL, Pan Z, Ekhtari N, et al. Capability Assessment and Performance Metrics for the Titan Multispectral Mapping Lidar. Remote Sens. 2016 Nov;8(11):936.

2. Chase AF, Chase DZ, Fisher CT, Leisz SJ, Weishampel JF. Geospatial Revolution and Remote Sensing LiDAR in Mesoamerican Archaeology. Proc Natl Acad Sci U S A. 2012 Aug 7;109(32):12916–21.

3. Chase AF, Reese-Taylor K, Fernandez-Diaz JC, Chase DZ. Progression and issues in the Mesoamerican geospatial revolution: an introduction. Advances in Archaeological Practice. 2016;4(3):219–31.

4. Evans DH, Fletcher RJ, Pottier C, Chevance J-B, Soutif D, Tan BS, et al. Uncovering Archaeological Landscapes at Angkor Using Lidar. Proc Natl Acad Sci. 2013;110(31):12595–600.

5. Stanton TW, Ardren T, Barth NC, Fernandez-Diaz JC, Rohrer P, Meyer D, et al. 'Structure' density, area, and volume as complementary tools to understand Maya Settlement: An analysis of lidar data along the great road between Coba and Yaxuna. J Archaeol Sci: Reports. 2020 Feb 1;29:102178.

6. ASPRS. LAS Specification 1.4 - R14. In: 2 LAS Format Definition. Bethesda, MD: American Society for Photogrammetry and Remote Sensing; 2011.

7. Fernandez-Diaz JC, Carter WE, Shrestha RL, Glennie CL. Now You See It… Now You Don't: Understanding Airborne Mapping LiDAR Collection and Data Product Generation for Archaeological Research in Mesoamerica. Remote Sens. 2014 Oct;6(10):9951–10001.

8. Yoëli P. The Mechanisation of Analytical Hill Shading. Cartogr J. 1967 Dec 1;4(2):82–8.

9. Devereux BJ, Amable GS, Crow P. Visualisation of LiDAR Terrain Models for Archaeological Feature Detection. Antiquity. 2008;82(316):470–9.

10. McCoy MD, Asner GP, Graves MW. Airborne Lidar Survey of Irrigated Agricultural Landscapes: An Application of the Slope Contrast Method. J Archaeol Sci. 2011 Sep 1;38(9):2141–54.

11. Chase ASZ. Beyond Elite Control: Residential Reservoirs at Caracol, Belize. WIREs Water. 2016;3(6):885–97.

12. Hesse R. LiDAR-Derived Local Relief Models – a New Tool for Archaeological Prospection. Archaeol Prospect. 2010;17(2):67–72.

13. Kokalj Z, Zaksek K, Ostir K. Application of Sky-View Factor for the Visualisation of Historic Landscape Features in Lidar-Derived Relief Models. Antiquity. 2011;85(327):263–73.

14. Zakšek K, Oštir K, Kokalj Ž. Sky-View Factor as a Relief Visualization Technique. Remote Sens. 2011 Feb 23;3(2):398–415.

15. Kokalj Ž, Somrak M. Why Not a Single Image? Combining Visualizations to Facilitate Fieldwork and On-Screen Mapping. Remote Sensing. 2019 Jan;11(7):747.

13. ESRI, ArcGIS Online | Cloud-Based GIS Mapping Software. *ESRI,* https://www.esri.com/en-us/arcgis/products/arcgis-online/overview (July 6, 2020).

14. ESRI, ArcGIS Collector | Capture Field Data - Data Collection App. *ESRI,* https://www.esri.com/en-us/arcgis/products/arcgis-collector/overview (July 6, 2020).

15. WhatsApp, WhatsApp. *WhatsApp.com*, https://www.whatsapp.com/ (July 6, 2020).

19. Sanders WT. The Cultural Ecology of the Teotihuacan Valley: A Preliminary Report of the Results of the Teotihuacan Valley Project. University Park, Pennsylvania: Pennsylvania State University; 1965. x, 209, [9] p. p.

20. Inomata T, Triadan D, Pinzón F, Burham M, Ranchos JL, Aoyama K, et al. Archaeological application of airborne LiDAR to examine social changes in the Ceibal region of the Maya lowlands. PLoS One [Internet]. 2018 Feb 21 [cited 2020 Apr 14];13(2). Available from: https://www.ncbi.nlm.nih.gov/pmc/articles/PMC5821443/

18. CONABIO, Gobierno de Mexico, Data from "Portal de Información Geográfica - CONABIO". Avaiable at http://www.conabio.gob.mx/informacion/gis/?vns=gis_root/dipol/dpotras/manzmge15gw (2020) (July 20, 2020).

19. Secretaría de Desarrollo Agrario, Territorial y Urbano, Data from "Gestión digital del territorio: Catálogo de Información Registral y Catastral". Available at https://datos.gob.mx/busca/dataset/gestion-digital-del-territorio-catalogo-de-informacion-registral-y-catastral (July 13, 2020).

20. A. Buckley, Getting better vectors from your rasters with ArcScan. *ArcGIS Blog,* https://www.esri.com/arcgis-blog/products/product/mapping/getting-better-vectors-from-your-rasters-with-arcscan/ (2008) (July 6, 2020).

24. Krivoruchko K. Empirical bayesian kriging. Esri: Redlands, CA, USA. 2012;Fall:6–10.
