# Supplementary material for: Humans as geomorphic agents: Lidar detection of the past, present and future of the Teotihuacan Valley, Mexico
Source: PLoS One. 2021 Sep 20;16(9):e0257550. doi: 10.1371/journal.pone.0257550 (PMC8452071; doi:10.1371/journal.pone.0257550)
Supplement: Attachment — Submitted filename: Response to Reviewers.docx [file pone.0257550.s003.docx]

**Reviewer #1 Comments:**

We thank Reviewer #1 for their positive review and minor edits and suggestion. All wording comments have been addressed as indicated by the change tracking system. Below we elaborate on how we have taken into consideration each point.

1. Title change suggestion from “Lidar detection of humans as geomorphic agents: The past, present and future of the Teotihuacan Valley, Mexico” to “Humans as Geomorphic Agents: Lidar Detection of the Past, Present, and Future of the Teotihuacan Valley, Mexico” has been made as suggested by the reviewer.
2. Reviewer requested we remove the dangling participle. We have changed sentence to “As humans are the primary geomorphic agents on the landscape, it is essential to assess the magnitude, chronological span, and future effects of artificial ground that is expanding under modern urbanization at an alarming rate.”
3. Reviewer has suggested we change repetition of word landscapes. We have changed the text to, “We argue humans have been primary geomorphic agents of landscapes since the rise of early urbanism that continue to structure our everyday lives.”
4. We have put the degree sign after 15.5.
5. Since the state had collapsed, we have clarified the sentence to “the legacy of the Teotihuacan’s influence continued both in…” as requested by the reviewer.
6. Table 3 and elsewhere – The reviewer mentions that because the term Ciudadela is so problematic—the structure is not actually a citadel— he/she suggested using the Spanish label (Ciudadela) rather than Citadel. We have fixed all mention of the Citadel to Ciudadela in the text and figures.
7. The only content-related suggestion was a cautionary note about comparing the Moon Pyramid volume with the Plaza of the Columns Complex volume because the former is a monument while the latter is an entire complex including basal platforms and residential complexes. Interestingly, despite this, Plaza of the Columns Complex volume is still lower than the Moon Pyramid.

The reason we have not included other flanking platforms is because we only have bedrock layer information for the Moon Pyramid itself, while our excavations at the Plaza of the Columns provided bedrock data for the entire complex. In addition, we cite the existing literature of volume calculations, which only provided volume estimates for the Moon Pyramid itself. Our hope is to create a volume estimate for the entire complex as we expand our database of known bedrock elevations. As stated in the text, these volume calculations are the beginning of a more accurate volume calculation of the ceremonial core at large, as we state, “Here we highlight some of the most conspicuous examples, but as more excavation data is gathered into the three-dimensional database, we will be able to more precisely calculate the volume of artificial ground across the valley.”

To avoid implying that these volume estimates are directly comparable, we have added the sentence, “This volume calculation does not include the platforms that comprise the Moon Plaza Complex and the parameter wall surrounding the Sun Pyramid, which would further enlarge their volumes.”

**Reviewer #2 Comments**

Reviewer #2 Comments: His/her concise and positive review only requested clarification concerning whether the Sanders survey data records of unpublished materials at Penn State were integrated into our map. We have modified the text to satisfy this clarification, “Two projects provided robust survey data across the entire lidar area, and we have georeferenced many of their published survey plans [11,12]. We are in the process of integrating the unpublished data from the Pennsylvania State archives.”
